# The feasibility and acceptability of screening for hypertension in private drug retail outlets: a pilot study in Mwanza region, Tanzania

**DOI:** 10.1093/inthealth/ihw023

**Published:** 2016-09-27

**Authors:** Denna Michael, Dotto Kezakubi, Adinan Juma, Jim Todd, Hugh Reyburn, Jenny Renju

**Affiliations:** a National Institute of Medical Research, Isamilo Road, P.O. Box 1462, Mwanza, Tanzania; b Kilimanjaro Christian Medical University College (Tumaini University), Department of Epidemiology and Biostatistics, P.O. Box 2240, Moshi, Tanzania; c The London School of Hygiene and Tropical Medicine, Keppel Street, London WC1E 7HT, UK

**Keywords:** Drug retail outlets, Hypertension, Pharmacies, Public–private partnerships, Screening, Tanzania

## Abstract

**Background:**

Hypertension is a major contributor to ill health in sub-Saharan Africa. Developing countries need to increase access for screening. This study assesses the feasibility and acceptability of using private sector drug retail outlets to screen for hypertension in Mwanza region, Tanzania.

**Methods:**

A pilot study took place in eight drug retail outlets from August 2013 to February 2014. Customers ≥18 years were invited for screening. Socio-demographic characteristics, hypertension knowledge, hypertension screening and treatment history were collected. Subjects with systolic blood pressure over 140 mmHg were referred for follow up. Referral slips captured attendance. Mystery client visits and follow up phone calls were conducted to assess service quality.

**Results:**

A total of 971 customers were screened, one person refused; 109 (11.2%) had blood pressure over 140/90 mmHg and were referred for ongoing assessment; 85/109 (78.0%) were newly diagnosed. Customers reported that the service was acceptable. Service providers were able to follow the protocol. Only 18/85 (21%) newly diagnosed participants visited the referral clinic within two weeks.

**Conclusions:**

Blood pressure screening was feasible and acceptable to customers of private drug retail outlets. However many who were referred failed to attend at a referral centre and further research is needed in this area.

## Introduction

Hypertension is a major contributor to ill health and premature mortality globally.^1–5^ There is now strong evidence that hypertension is increasingly common in sub-Saharan Africa and community-based surveys suggest that the prevalence of hypertension is approaching that found in high-income countries.^6^ Studies conducted in rural Tanzania, Ghana and Cameroon have found prevalence of hypertension among adults over the age of 40 years to vary between 20 and 30%.^2,7–9^ Despite this relatively high prevalence, rates of detection, treatment and control of hypertension in sub-Saharan Africa are generally low.^9–18^

In developed countries community-based screening by non-physicians or non-clinicians has been shown to have added advantages over routine screening for detecting hypertension in individuals outside health care settings.^10,11^ Self-screening, as a variant of community based screening for hypertension, is now practiced as pharmacy-based screening.^10,11^ However there are no studies of a similar approach in Africa.

This study was conducted in two districts of Mwanza region, Tanzania to determine whether private drug retail outlets would be a feasible and acceptable venue to screen individuals for hypertension and subsequently direct them to proper care and treatment.

## Methods

### Setting

The study was conducted in a rural (Magu: population of 299 759 [2012 Census]) and urban district (Nyamagana: population of 363 452 [2012 Census]) in Mwanza region, in the Northwest of Tanzania. The two districts were purposively selected to ensure representation of both urban and rural communities and accessibility from the research centre.

### Sampling and sample size

All private/government health facilities offering services for hypertension in the study area and a list of all registered private drug dispensing outlets (stratified by type) were provided by the district medical officer. The list of the private drug dispensing outlets was further refined to include only those facilities that were situated within the catchment area (5 km radius or within 30 minutes walking distance) of the selected health facilities. The sampling frame from which outlets were drawn included four pharmacies and 14 accredited drug dispensing outlets (ADDOs) in Nyamagana district and two pharmacies and 10 ADDOs in Magu district. One pharmacy and three ADDOs were randomly selected from the list from each district. All adults (aged ≥18 years) visiting selected private drug retail outlets from October 2013 to January 2014 were eligible to participate in the study*.* We made a conservative estimate that each private drug retail outlet could receive between 5 and 15 visitors a day of which 80% could be eligible and 50% could be new clients. We assumed that across the eight outlets we would be able to recruit 500 individuals per month. In two months, an approximate sample of 1000 study participants would be enough to understand the feasibility and acceptability of the screening service.

### Data collection

The person in charge at each private drug retail outlet was trained on how to guide customers to take their own blood pressure using a digital, semi-automated blood pressure monitor with advanced oscillometric measurement (Model UA-767, A&D Company Ltd, Tokyo, Japan.) The drug dispenser gathered information through face to face interview using a questionnaire. No financial incentive was given to pharmacists or their customers at the onset of this study out of concern that such an incentive would bias the data collection and not support the sustainability of the intervention. The questionnaire collected socio-demographic information, exposure to previous hypertension/blood pressure information, previous screening, diagnosis and/or treatment, telephone contact details and patient satisfaction. Data were recorded manually and verified weekly by a field supervisor. The person in charge was not supposed to diagnose or give medication for study participants found to have hypertension. After 5 minutes seated rest consenting adults took their first blood pressure reading, this was repeated for the second reading. Only participants with high blood pressure in both readings were classified as hypertension cases and referred to the district hospital. The second reading was used in the analysis. All participants should have been given pamphlets containing information on hypertension prevention and risk factors as well as emphasizing the need for those with hypertension to go for follow up.

At the outpatient department of the selected hospitals, a dedicated nurse was trained to receive referred participants and to complete the study specific referral slip. The nurse received 1000 Tanzanian Shillings (approximately US$0.5) for each correctly completed referral slip.

Four mystery clients (2 male, 2 female, all over 30 years) were selected and trained on the study design and objectives, how to enter the facility, how to ask for the service should it not have been offered and how to rate the service. Their visits occurred at random intervals throughout the study period on different days. They entered the facilities for an unrelated service and allowed the service provider time to offer the service; should no service be offered they made a passing inquiry about the blood pressure poster and waited to see if they were then offered the service. After leaving the facility the research assistant administered a short questionnaire. This component provided verification on quality, fidelity to the study protocol, and how much time was utilized identifying, enrolling, consenting and instructing participants into the study.

Telephone interviews were conducted with all participants with hypertension and every fifth form received from each facility with a normal reading. A semi-structured questionnaire was used to investigate the service user perceptions of the service and was conducted 2 weeks after the blood pressure reading to allow for attendance at the health facility.

Follow up phone calls were used to verify referral slips. Bi-weekly site visits were made by study personnel and real-time reports were produced to ensure that pharmacists, drug dispensers, and personnel in the referral sites were collecting the data properly.

### Data management and statistical analysis

Data were double-entered, cleaned and validated using EpiData version 3.1 (The EpiData Association, Odense, Denmark) and exported to STATA 13 (Stata Corp, College Station, TX, USA). Hypertension status was classified using the 7th report of the Joint National Committee on Prevention, Detection, Evaluation, and Treatment of High Blood Pressure Joint National Commit criteria (JNC7 criteria).^12^ According to JNC7 criteria, hypertension was defined as blood pressure >140 mmHg and/or diastolic blood pressure >90 mmHg and/or currently taking any antihypertensive medication. Mystery client responses were grouped by district and then by drug dispensing outlet to understand outlet specific situations. Open ended responses were post coded and described qualitatively; quotations were used to highlight key findings.

### Ethical issues

Written informed consent was obtained from each study participant before any data was collected or blood pressure measurements were taken. An impartial witness was present for the consent of illiterate participants, who then used a thumb print to illustrate consent.

## Results

**Figure 1.**
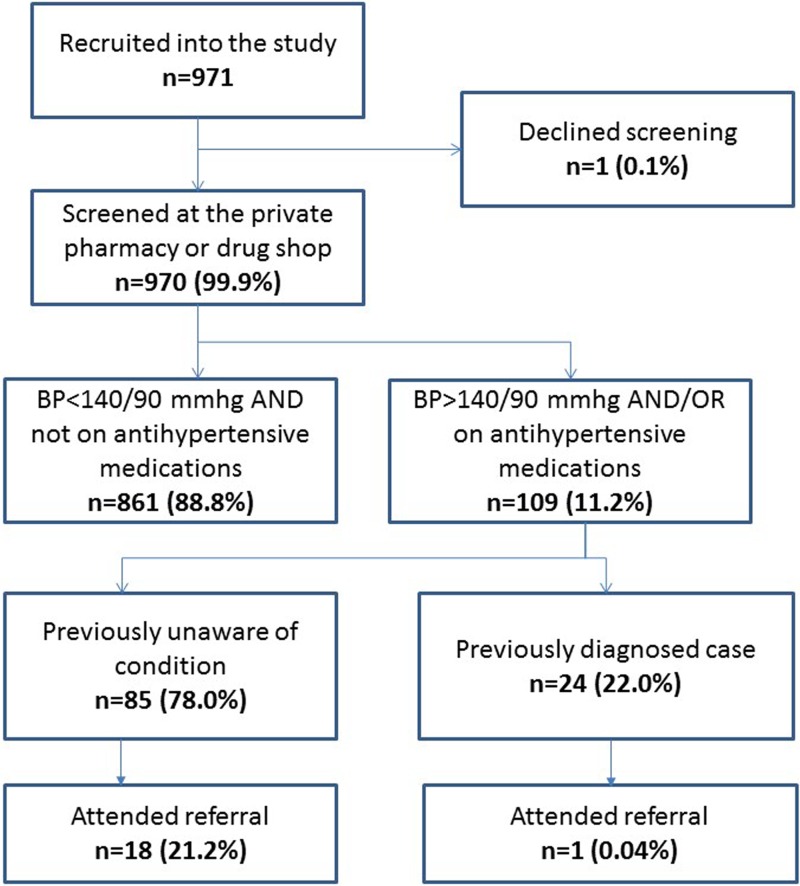
Flow chart showing the study participants from recruitment, through screening and referral to attendance at referral.

A total of 971 study participants were approached to participate in the study, of whom, 970 consented and were screened for high blood pressure and recruited into the study. Only one person declined to participate, for unknown reasons. A total 701 (72.2%) were recruited from six small drug retails outlets whilst 269 (21.8%) were from the large pharmacies. The majority of the participants (529,54.6%) resided in the rural district of Magu at the time of recruitment. Out of 970 study participants 494 (51.0%) were males and 664 (68.4%) were below 40 years of age. The mean age for all study participants was 35 years (SD 13.2). Characteristics of subjects with and without hypertension are show in Table 1. The majority of participants reported to have never heard of hypertension (639, 65.9%) or previously measured their blood pressure (819, 84.4%).The majority of those screened (861, 88.8%) recorded normal blood pressure readings (see Table 2) and were not previously on any antihypertensive drugs. The overall prevalence of hypertension amongst the study participants was 109 (11.2%) of whom 85 (78.0%) were newly diagnosed (see Figure 1).

**Table1.** Participants' characteristics and factors associated with hypertension, (n=970)

Variables	Total	%	Hypertension	%
All	970		109	11.2
Age category				
18–29	426	43.9	13	11.9
30–39	238	24.5	23	21.1
40–49	147	15.2	26	23.9
>50	159	16.4	47	43.1
Sex				
Female	467	48.1	59	54.1
Male	494	50.9	48	44.0
Missing	9	0.9	2	1.8
Marital status				
Single	317	32.7	18	16.5
Married	596	61.4	83	76.1
Divorced	51	5.3	7	6.4
Missing	6	0.6	1	0.9
Occupation				
Peasant	371	38.2	45	41.3
Employed	258	26.6	26	23.9
Business	334	34.4	36	33.0
Missing	7	0.7	2	1.8
Education				
None	45	4.6	9	8.3
Primary	520	53.6	68	62.4
Secondary	296	30.5	24	22.0
Tertiary	101	10.4	7	6.4
Missing	8	0.8	1	0.9
District				
Urban	441	45.5	42	38.5
BP Measured before				
Yes	144	14.8	24	22.0
No	819	84.4	84	77.1
Missing	7	0.7	1	0.01
Heard of hypertension				
Yes	322	33.2	40	36.7
No	639	65.9	68	62.4
Missing	9	0.9	1	0.9
Treated for hypertension				
Yes	67	6.9	12	11.0
No	890	91.8	96	88.1
Missing	13	1.3	1	0.9
Drug outlet				
Pharmacy	269	27.7	24	22.0
ADDO	701	72.3	85	78.0

ADDO: accredited drug dispensing outlets.

**Table 2.** Distribution of mean systolic and diastolic blood pressure by hypertension status and socio-demographic characteristics

Residence	Sex	Age group	Number	Systolic blood pressure		Diastolic blood pressure	
				Mean (SD)	95% CI	Mean (SD)	95% CI
Urban district							
	Male	18–29	77	123.5 (12.2)	121–126	78.5 (10.8)	76–81
		30–39	56	126.1 (14.7)	122–130	81.6 (11.8)	78–85
		40–49	27	132.0 (23.9)	123–141	82.6 (13.0)	78–88
		>49	41	152.1 (28.9)	143–161	88.2 (13.8)	84–93
		All	201	131.2 (21.8)	128–134	81.9 (12.5)	80–84
	Female	18–29	111	118.8 (13.6)	116–121	80.5 (10.3)	79–82
		30–39	68	121.2 (12.6)	118–124	83.6 (9.4)	81–86
		40–49	28	130.8 (23.7)	122–140	88.0 (17.0)	81–95
		>49	26	147.2 (35.1)	133–161	88.3 (15.6)	82–95
		All	233	124.1 (20.3	121–127	83.2 (12.0)	82–85
	Total		440	127.5 (21.3)	125–129	82.7 (12.3)	82–84
							
Rural district							
	Male	18–29	127	123.4 (18.0)	120–127	76.3 (10.9)	74–78
		30–39	59	126.5 (17.1)	122–131	82.4 (12.6)	79–86
		40–49	51	129.0 (19.6)	124–135	85.0 (14.2)	81–89
		>49	55	137.2 (18.9)	132–142	85.0 (12.2)	82–88
		All	292	127.6 (18.9)	125–130	80.7 (12.7)	79–82
	Female	18–29	106	117.8 (17.8)	114–121	75.2 (12.8)	73–78
		30–39	52	130.0 (24.2)	123–137	85.0 (16.8)	80–90
		40–49	39	131.6 (17.8)	126–137	86.0 (14.3)	81–91
		>49	36	143.2 (29.9)	133–153	90.1 (15.7)	85–95
		All	233	126.7 (23.3)	124–130	81.5 (15.6)	79–84
	Total		528	127.3	126–129	81.1 (14.1)	80–82
Combined							
	Male	18–29	204	123.4 (16.0)	121–126	77.1 (10.9)	76–79
		30–39	115	126.5 (15.9)	123–129	82.0 (12.2)	80–84
		40–49	78	130.1 (21.1)	125–135	84.0 (13.8)	81–87
		>49	96	143.5 (24.6)	139–149	86.4 (13.0)	84–89
		All	493	129.1 (20.2)	127–131	81.2 (12.6)	80–82
	Female	18–29	217	118.3 (15.8)	116–120	77.9 (11.9)	76–79
		30–39	120	125.0 (19.0)	122–128	84.2 (13.1)	82–87
		40–49	67	131.2 (20.3)	126–136	86.8 (15.4)	83–91
		>49	62	144.8 (32.0)	137–153	89.4 (15.6)	85–93
		All	466	125.4 (21.9)	123–127	82.3 (14.0)	81–84
	Total		968	127.4 (21.2)	126–129	81.8 (13.3)	81–83

### Referral attendance and challenges of the screening program

Only 18/85 (21%) of the newly diagnosed and only one of the 24 previously diagnosed cases attended referral within two weeks of their referral. Of the 18, nine were aged 50 years and above, 10 were male, 13 were married and 16 had a higher education level.

### Quality and acceptability of the service

A total of 340 study participants were selected to be contacted by telephone after being screened: only 157 (46.2%) were successfully reached and completed a questionnaire. No respondent reported any problems with the service provided and all were able to take their blood pressure readings. The majority (133/157, 84.7%) reported they would recommend the service to someone else. This finding did not vary by age, gender or blood pressure reading.

Thirty two mystery client visits took place (see Table 3) across eight facilities (four per facility). Service provision took between 3 and 75 minutes with a mean time of 20 minutes and over half of the visits taking less than 18 minutes. The majority of providers offered the service without the need for prompting (25/32, 78%) and were able to fully explain the study (22/32; 69%). Findings from one facility showed that each mystery client reported that the provider was unable to explain the testing process and this was the same facility in which the provider failed to explain the study completely. Mystery clients from these facilities explained that instead of being told how to measure their own blood pressure the provider simply took the measurement themselves (in seven out of 32 facilities). In one facility the blood pressure machine was being stored somewhere else and the provider had to leave the facility to locate the machine (on the insistence of the mystery client).

**Table 3.** Responses collected from the interviews that followed the mystery client visits

Variables	Total	%
All	32	
Magu	16	50
Nyamagana	16	50
How were services offered		
Providers offered the service to the mystery client without any prompting	25	78
Able to explain the study		
The provider was able to fully explain the study to the mystery client	22	69
Explanation of the testing process		
The provide was able to fully explain the testing process to the mystery client	24	75
Able to answer all the questions		
The provider was able to answer all the questions posed by the mystery client	32	100
Information provided		
The provider gave an information leaflet to the mystery client	9	28
Missing	2	6
How the mystery client rated the service		
Friendly and helpful explaining steps	27	84
Friendly but too busy to help	4	13
Too busy and not interested in helping	0	0
Not busy but not interested in helping	1	3
Time taken to provide the service		
Mean time, minutes	19.8	
Median time, minutes	18.0	
Range, minutes	3–75	

All mystery clients reported that their questions were answered fully when they asked them, however the majority (21/30, 70%) reported to have not been given any leaflet.

The majority of mystery client visits recorded that the service provider was friendly and helpful in explaining the steps (27/32, 84%). In addition to this four of the providers actively encouraged the mystery clients to invite others to come for the service. Of the four mystery client visits that said the service providers were friendly but too busy to help (4/32, 13%), three were from the same facility in which they reported that the study and testing process was not well explained.

## Discussion

This pilot feasibility study has shown that screening for hypertension at the private drug retail outlets was acceptable by users of the service and possible to conduct by drug dispensers and therefore could be feasible to offer on a larger scale. Only one person refused the service (for unknown reasons), all others were happy to screen themselves and would recommend the service to others. The main diversion from the protocol involved the service providers taking the measurement themselves rather than instructing the clients to take their own; therefore not always being offered as self-screening. Some medical personnel were concerned that measurement by the service provider would be seen, by some participants, as a diagnosis of hypertension. Very low numbers of participants had ever heard of hypertension or ever tested, highlighting a low level of awareness of this rapidly growing health problem in this region and suggestive of a large unmet need for screening services. The screening identified a reasonable number of people with hypertension (109/970, 11.2%) who were referred for further follow up. However very few (19/109, 17.4%) attended a referral site within two weeks of the referral.

Our study supports a number of other studies in sub-Saharan Africa that highlight the worrying burden of hypertension in this region of the world.^2,3,6,7,13^ Our findings support other studies which show that very few individuals with hypertension were previously diagnosed and fewer had their blood pressure under control.^7,14^ Despite the high burden of disease, our study also showed that, most people lacked knowledge and awareness which could have negatively impacted on their timely access to diagnosis and care. These findings suggest that public health screening at private drug retail outlets, both ADDO and pharmacies, might be an acceptable and highly needed service to compliment the routine healthcare delivery system.^15^

However our study documented low referral uptake. Less than a quarter of study participants with high blood pressure readings attended their referrals. Although there was a local referral system set up between selected private drug retail outlets and nearby government-run hospital, some people could have gone to other hospitals away from the study area, others could have gone after the two week study follow up period (such services are most typically offered at tertiary level facilities). Uptake of hypertensive health services had been reported to be low elsewhere in Tanzania.^15^ In a population-based study in Dar es salaam, only 34% of those previously diagnosed with hypertension were reported to have attended a formal healthcare provider within 12 months.^15^ Most studies elsewhere in sub-Saharan Africa have documented low levels of awareness which resulted in poor linkage to healthcare providers, and control, which can adversely affect treatment outcomes.^2^ More research and intervention development is needed to design better mechanisms for facilitating a timely referral to treatment and management services.

Our study was a small pilot study and therefore has a number of limitations. Our study participants were recruited based on their willingness to participate, therefore our responses could be affected by volunteer bias, but only one person declined to take part in the study; a telling result in itself in terms of acceptance of the service. The majority of study participants were below 40 years of age possibly affecting the mean systolic blood pressure results, that are less likely to rise in this age group.^6^ The intervention did not include any community based advertisements of the service, which could help to attract different demographic groups (i.e., older people). In our study we were unable to calculate accurate prevalence estimates or assess community level understanding and awareness of hypertension; a community based survey would be needed to provide better estimates but was out of the scope of this study. For the follow up phone calls only 46% of the service users were reachable on the phone numbers they gave during screening. This was a much lower number than we expected. Our study suggests that relying on a single mobile phone contact number per participant was inadequate and additional information is needed in order to maximize on mobile technology as part of any intervention.^16^ That being said, the sample reached included a representation of ages and over one third had hypertension, which was deemed sufficient to ascertain how they rated the service in terms of ease to conduct and whether they would recommend it to a friend. More research is needed to understand why some participants had their blood pressure measured by the pharmacist attendant whilst others were left to measure themselves. The study was only able to capture a small number of those attending referral and therefore we did not have the power to investigate the factors associated with successful referrals. A larger sample size and broader range of socio-demographic and health system risk factors are needed to investigate explanations for ineffective referrals among hypertensive patients. Our study did not collect data to analyze the time from diagnosis to attendance of the referral. Most cardiovascular complications of hypertension are caused by uncontrolled higher levels of diastolic and/or systolic blood pressure for long periods of time. Greater focus should be on understanding how fast people with hypertension will navigate to reach healthcare just after their diagnosis. Time taken from initial diagnosis to blood pressure control may also be required in future research.

### Conclusions

The current pilot study suggests that it might be feasible and acceptable to implement a hypertension screening intervention program in private drug retail outlets in Tanzanian. Data from projections and models have shown that the growing disease burden due to chronic disease will cause most governments to lose tens of billions of dollars from their national income. Therefore, governments need to promote access to preventive and treatment services.^1^ Low cost, scalable and equitable interventions are needed to improve access to prevention, identification and treatment services for chronic diseases including hypertension.^1^
